# Preparation, Structure and Characterization of Polymer/Cement Composites—3rd Edition

**DOI:** 10.3390/polym17222980

**Published:** 2025-11-09

**Authors:** Bowen Guan

**Affiliations:** School of Materials Science and Engineering, Chang’an University, Xi’an 710061, China; bguan@chd.edu.cn

Polymer/cement composites and related materials have garnered substantial attention in civil engineering, primarily due to their enhanced properties compared to traditional cement-based materials. With the rapid advancement in polymer/cement composite technology and extensive research on the relationship between material structure and properties, the utilization of these composites has witnessed a significant surge. Consequently, research in this domain has become a focal point both in China and globally. These composites have found applications in various civil engineering sectors.

In soil stabilization, polymer-based techniques have proven effective. Wei et al. conducted a predictive analysis of the mechanical properties of biopolymer-fiber-reinforced composite-stabilized soil using genetic algorithm-optimized back propagation neural networks [1]. Chunqiu Xia et al. studied the effect of acrylate emulsion on the mechanical and microscopic properties of straw fiber-reinforced cement-magnesium slag stabilized soil [2]. Moreover, polymers, as long-chain molecules, can significantly enhance soil’s physical and chemical properties, including strength, stability, and water retention capacity.

Engineered cementitious composites have also benefited from polymer additions. Mohammed A. Albadrani investigated the influence of nanosilica and PVA fibers on the mechanical and deformation behavior of such composites [3]. In the context of cement-polymer compatibility, Yahya Kaya et al. experimentally and modeled the assessment of parameters affecting grinding aid-containing cement-PCE compatibility using CRA, MARS, and AOMA-ANN methods [4]. Furthermore, Wang et al. explored the effects of C-S-H seed prepared by wet grinding on the properties of cement containing large amounts of silica fume [5].

Polymer modifications have significantly impacted asphalt materials. Research on the microscopic aging characteristics of asphalt binders has been conducted using advanced techniques like atomic force microscopy [6]. Mustafa Alas et al. analyzed the optimum performance of polymer and polymer-nanocomposite-modified asphalt using multicriteria decision analysis [7]. Zhang et al. characterized the tensile properties and nanoscale phase structures of modified asphalts and their aging behavior [8]. Additionally, Ming Wang et al. recognized and characterized nanoscale phases through modulus mapping of asphalt film in pavement mixture cores [9]. Li et al. investigated the effects of silicone rubber on the rheological properties and aging characteristics of asphalt binders [10].

In concrete technology, polymers have revolutionized material performance. Metin Tuncer et al. experimentally investigated the durability properties of polymer-coated pumice aggregate lightweight concretes [11]. The tensile properties of concrete can also be improved through polymer additions, as seen in the work of various researchers. Furthermore, Tri N. M. Nguyen et al. studied the changes in the inner-structure and mechanical strength of composite cement materials reinforced with silica-carbon nanotube-nylon 66 electrospun nanofibers [12].

Polymer modifications have also been applied to asphalt mortars. Zhang et al. researched the performance of bamboo bark fiber asphalt mortar modified with surface-grafted nano-SiO_2_ [13]. Such modifications enhance the mortar’s properties, making it more suitable for specific applications.

In the realm of reinforced concrete, the use of fiber-reinforced polymers (FRP) has gained prominence. Mahmood Haris et al. reviewed the state-of-the-art on strengthening reinforced concrete members using FRP, evaluating fire performance, challenges, and future research directions [14]. This comprehensive review highlights the potential and challenges associated with FRP in concrete structures.

In conclusion, polymer/cement composites and related materials have emerged as promising solutions in civil engineering, offering superior properties and performance compared to traditional materials. With ongoing research and development, the application of these composites is expected to expand further, driving innovation and sustainability in the construction industry.
